# Insights into roles of METTL14 in tumors

**DOI:** 10.1111/cpr.13168

**Published:** 2021-12-13

**Authors:** Xin Liu, Yuping Du, Zhenghao Huang, Honglei Qin, Jingwen Chen, Yang Zhao

**Affiliations:** ^1^ Department of Obstetrics and Gynecology Department of Gynecologic Oncology Research Office Key Laboratory for Major Obstetric Diseases of Guangdong Province The Third Affiliated Hospital of Guangzhou Medical University Guangzhou China

**Keywords:** cancer, m6A, METTL14, non‐coding RNA, RNA modification

## Abstract

N6‐Methyladenosine (m6A) is considered the most common and endogenous modification of eukaryotic RNAs. Highly conserved in many species, m6A regulates RNA metabolism, cell differentiation, cell circadian rhythm, and cell cycle; it also responds to endogenous and exogenous stimuli and is associated with the development of tumors. The m6A methyltransferase complex (MTC) regulates the m6A modification of transcripts and involves two components, methyltransferase‐like enzyme 3 (METTL3) and methyltransferase‐like enzyme 14 (METTL14), and other auxiliary regulatory distinct components. Though with no catalytic effect, METTL14 serves as an RNA‐binding scaffold in MTC, promotes RNA substrate recognition, activates, and escalates the catalytic capability of METTL3, thus accounting for a pivotal member of the complex. It was reported that METTL14 regulates tumor proliferation, metastasis, and self‐renewal, and plays a part in tumorigenesis, tumor progression, and other processes. The present work is a review of the role of METTL14 both as a tumor suppressor and a tumor promoter in the oncogenesis and progression of various tumors, as well as the potential molecular mechanisms.

## Graphical abstract

Roles of METTL14 in oncogenesis and development. The abnormal expression of METTL14, an m6A modification editor, affects tumor cell proliferation, metastasis, tumor stem cell self‐renewal, and chemotherapy resistance.

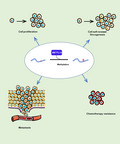

## 1 INTRODUCTION

**FIGURE 1:**
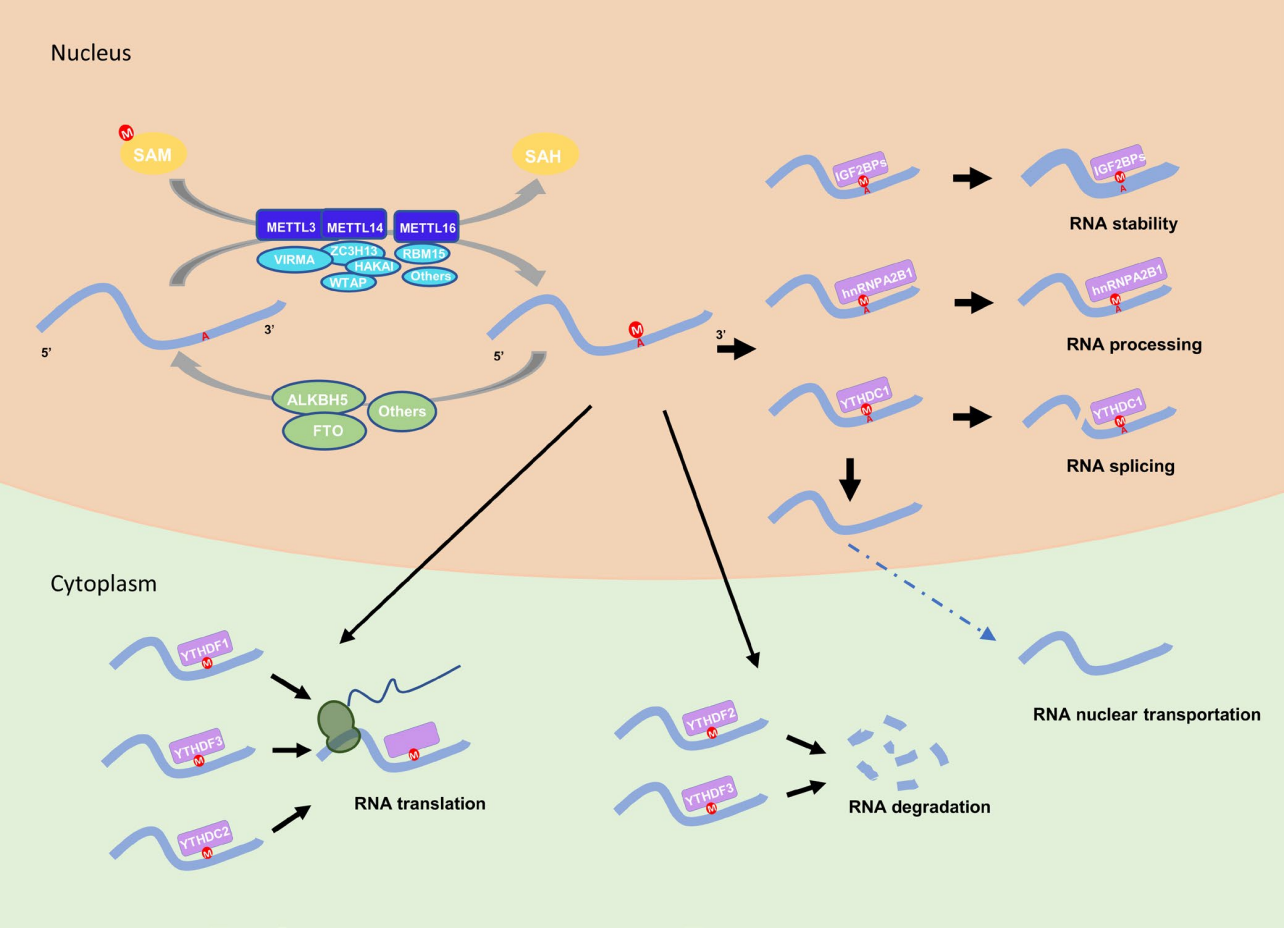
RNA m6A modification is dynamically and reversibly co‐regulated by RNA m6A modification machineries EEE. RNA m6A modification machineries EEE are consisted of Editors, Erasers, and Effectors. M6A is responsible for cell differentiation, cell circadian rhythm, cell cycle, and cell stress response by affecting various aspects of RNA metabolism, such as mRNA maturation, nucleus transport, RNA splicing, stability, and protein translation efficiency

Epigenesis refers to the heritable variation of gene functions under the condition that the DNA sequence does not change, which ultimately leads to the change in phenotype.^1^, ^2^ Epigenetics participates in and regulates multiple levels of information flow from DNA to RNA to protein.^3^, ^4^ Recent investigations have found that the epigenetic modification of RNA plays vital roles in biological processes.^5^ N6‐methyladenosine (m6A), as one of more than 170 RNA modifications observed so far in coding and non‐coding of RNAs, is the most common internal modification in mRNA,^6^, ^7^ and is almost universally presented in poly (A)+ RNA of all advanced eukaryotes.^8^, ^9^ In human cells, there are 2000 m6A peaks in beyond 7000 mRNA and 300 non‐coding RNA transcripts.^10^ It was found that m6A is not randomly distributed on mRNA, but mainly clustered in long introns and stop codons near the 3′UTR region.^11^ Under similar physiological conditions, highly conserved m6A peaks have been observed in mouse and human transcriptomes, revealing significant correlation between m6A abundance and functions of specific genes.^10^ In mutation analysis, RRm6ACH has been defined as a consensus m6A sequence, where R = G/A (G > A) and H = U/A/C (U > A > C).^12^ In the early 1970s, m6A modification was first detected in mammalian cells, but due to the constraints of the research conditions and technology at that time, the functional significance of this modification was not clarified. The true biological significance of m6A was not disclosed until 2011 when it was discovered that fat mass and obesity‐associated (FTO) protein reversibly inhibited the m6A level.^13^ Meanwhile, advancements in sequencing technology and quantitative mass spectrometry technology^10^, ^11^, ^14^ encouraged researchers to probe further into m6A modification. It was reported that m6A can regulate cell differentiation,^15^, ^16^ cell circadian rhythm,^17^ cell cycle,^18^, ^19^ and cell stress response^20^, ^21^ by participating in various aspects of RNA physiological processes, such as mRNA maturation,^22^, ^23^, ^24^ transport from nucleus to cytoplasm,^25^, ^26^ translation efficiency^21^, ^27^, ^28^ and stability^29^, ^30^ (Figure 1).

RNA m6A modification is co‐mediated by the modification machineries EEE (Editor, Eraser, and Effector).^31^ The modification Editor—multicomponent methyltransferase complex (MTC), positioned in the nuclear spot area, catalyzes the dislocation of methyl group to the certain adenine of the target RNA from S‐adenosylmethionine (SAM) and methylates the hydrogen atom on the adenine atom N6,^32^, ^33^ by recruiting two predominant units, that is, methyltransferase‐like enzyme 3 (METTL3) and methyltransferase‐like enzyme 14 (METTL14), and with other auxiliary managing distinct units, such as Wilm's tumor‐1‐associated protein (WTAP), vir‐like m6A methyltransferase‐associated (VIRMA), KIAA1429, Hakai, RNA‐binding motif protein 15 (RBM15), and methyltransferase‐like enzyme 16 (METTL16). The modification Erasers—FTO and alkB homolog 5 (ALKBH5), are both Fe (II)/α‐ketoglutarate‐dependent alkB dioxygenase family members, which perform as m6A erasers and catalyze the reversible oxidative demethylation of the m6A methyl group.^34^, ^35^ The messages embedded in the methyl group are decoded by a certain type of protein component called m6A Effectors, including YTH domain family proteins (YTHDC1/2 and YTHDF1/2/3), insulin‐like growth factor 2 mRNA‐binding protein (IGF2BP1/2/3), and heterogeneous ribonucleoprotein (hnRNPC and hnRNPG), which bind m6A to modulate the nuclear transportation, degradation, and steadiness of mRNAs, thereby determining the fate of the modified target RNA.^21^, ^36^, ^37^

Methyltransferase and its components can regulate cell cycle, cell growth, cell differentiation, cell apoptosis, and other cellular biological processes. The correlation between methyltransferase and tumors has become a new research focus. It has been found that m6A modification and methyltransferase are related to tumor augmentation, differentiation, tumor formation, infiltration, and metastasis. Recently, increasing proof has attested to the involvement of METTL14, an indispensable constituent of MTC, in cancer. Here, we review the role methyltransferase METTL14 in tumor occurrence and expansion.

## 2 STRUCTURE AND FUNCTIONS OF METTL14 IN MTC

**FIGURE 2:**
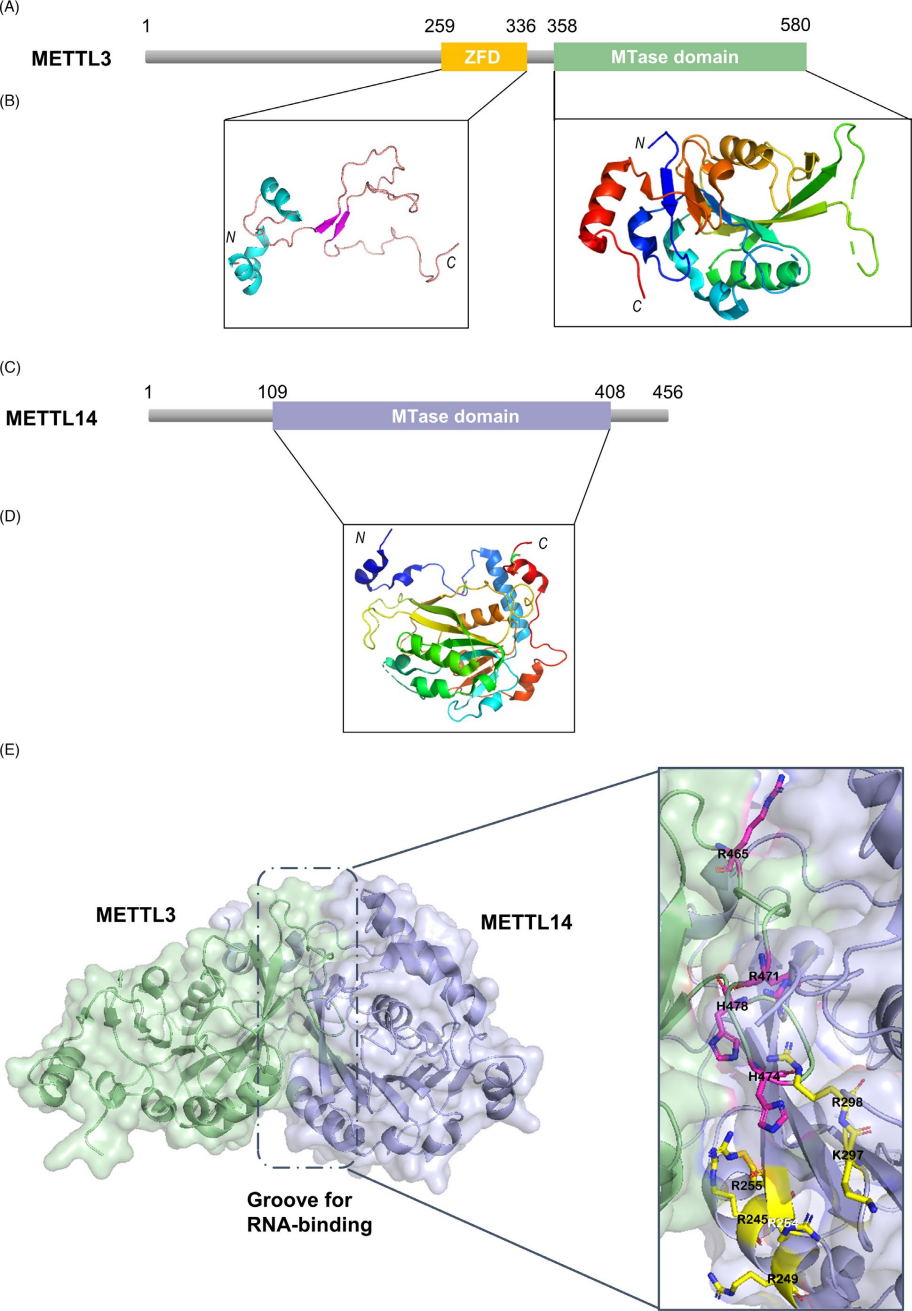
Structure and functional subunit of METTL14. (A) Schematic domain structure of METTL3. (B) Structure of the zinc finger domain (ZFD) (PDB ID: 5YZ9) and methyltransferase domain (MT‐A70) of METTL3 (PDB ID: 5L6D). (C) Schematic domain structure of METTL14. (D) Structure of the methyltransferase domain of METTL14 (PDB ID: 5IL0). (E) The RNA‐binding groove with positive charges between METTL3 and METTL14. All structure figures were prepared using PyMOL

In the last decade of the 20th century, Tuck^38^ isolated two methylase constituents of 200 kDa (MT‐A) and 800 kDa (MT‐B), from the nuclear extract of Hela cells, and identified one key methylase subunit 70 kDa, named METTL3 or MT‐A70, from MT‐A. This investigation marks a considerable breakthrough in the research of m6A methyltransferase. The METTL3 protein, which has 580 amino acids, is comprised of a zinc finger domain (ZFD) and a methyltransferase domain (MTD). The ZFD contains two tandem CCCH‐type zinc fingers (ZnF1 and ZnF2) connected by an anti‐parallel β‐sheet (Figure 2A,B), which is responsible for target recognition, specifically for binding to single‐stranded RNAs containing 5′‐GGACU‐3′ consensus sequence.^39^ Phylogenetic analysis revealed that METTL14, a homologue of METTL3 in the human genome, shares 43% homologous sequence with METTL3.^40^ The METTL14 gene is 30.06 kb in size, contains 11 exons, and is positioned on chromosome 4q26. This gene is expressed in many tissues at different developmental stages. As relevant research deepens, more knowledge about the METTL14 gene, including its expression products, structure, and functions, is revealed. The METTL14 protein has 456 amino acids and is predominantly composed of a MTD, while without a ZFD as the METTL3 protein does (Figure 2C,D). METTL14 and METTL3 form a compact and stable asymmetric heterodimer with a 1:1 stoichiometric ratio, and there are extensive hydrogen bond interactions between the two.^41^ X‐ray crystallography was adopted to inspect the structure of the MTD of the METTL3‐14 heterodimer. The MTD of METTL14 encompasses residues 110–404, while that of METTL13 consists of residues 358–580. The crystal structure of the heterodimer revealed dense presence of SAM in the catalytic cavity of METTL3; the MTD of METTL14, though similar to that of METTL3, has a closed catalytic cavity with no SAM binding sites, which indicates that METTL3 is the only catalytic subunit in the MTC, and METTL14 in the complex does not serve a role to catalyze methyl group transfer.^40^ The overall structure of MTD3 resembles that of Class I DNA N6‐adenine MTD. Nevertheless, neither MTD3 nor MTD14 possesses a target recognition domain (TRD) similar to DNA m6A, which appears as a substrate‐binding platform. Analysis of the surface electrostatic potential of the METTL3/METTL14 complex demonstrated that there is a groove with positive charges between METTL3 and METTL14. Ten positively charged residues, including R245, R249, R254, R255, K297, and R298 from METTL14, and R465, R471, H474, and H478 from METTL3, together shapes this groove (Figure 2E). In the case that the above residues from METTL14 in the groove are mutated, the activity of groove‐bound RNA decreases and the activity of methyltransferase drops,^42^ indicating that the groove formed by the positively charged residues of METTL3 and METTL14 may affect the binding of the complex to the RNA substrate. A recent study revealed that the C‐terminus of METTL14 was induced arginine methylation by binding to protein arginine methyltransferase 1 (PRMT1), which promotes the interaction of RNA substrates to METTL14, and enhances the RNA methylation catalytic capacity of the MTC and interaction with RNA polymerase II (RNAPII).^43^ Importantly, the level of m6A modification is dependent on arginine methylation of METTL14. Analysis of transcriptome m6A modification levels revealed that nearly 2000 m6A sites, which depend on arginine methylation of METTL14, distribute over 1000 genes involved in cellular physiological processes including DNA repair. Specifically, the m6A modification of DNA double‐stranded cross‐linking repair‐related genes relies on arginine methylation of METTL14, which improves the translation efficiency of these genes. In addition, the N‐terminus of METTL14 can discern and directly interact with histone 3 trimethylated at Lys 36 (H3K36me3). The combination promotes recruitment of the MTC to RNAP II nearby, revealing that the MTC exerts cotranscriptional selective deposition by moving to the site of immature RNA.^44^ Liu et al.^41^ reported that the catalytic activity of the METTL3‐METTL14 complex has considerably stronger catalytic activity than the METTL3 protein isolated in vitro. The above results indicate that though METTL3 plays a catalytic role in the MTC, its catalytic capability relies on METTL14. Though METTL14 itself has no catalytic effect, it acts as a premier RNA‐binding platform, which promotes the recognition to RNA substrates, activates, and escalates the methyltransferase activity of METTL3, and significantly improves the methylation efficiency of the MTC. The role of METTL14 in the METTL3‐METTL14 MTC is much like that of Dnmt3L in the DNA methyltransferase complex Dnmt3a‐Dnmt3L: Dnmt3L activates and enhances the methylation activity of the DNA methyltransferase complex by binding to Dnmt3a, but Dnmt3L itself has no methylation activity.^45^ Therefore, in general, METTL14 is similar to Dnmt3L in that they both contain MTD without catalytic activity, but activate and strengthen the methylation of the chaperone.

## 3 MODULATION OF METTL14 EXPRESSION

The abnormal expression of METTL14 in cancers and other diseases is triggered by multiple mechanisms. It was reported that METTL14 overexpression in acute myeloid leukemia is negatively regulated by SPI1 and mediates downstream targets, MYB and MYC, to accelerate acute myeloid leukemia (AML) oncogenesis.^46^ In breast cancer, aurora kinase A (AURKA) positively regulates METTL14 protein expression by inhibiting its ubiquitylation‐mediated degradation to elevate DROSHA m6A content to improve DROSHA mRNA stability in breast cancer stem‐like cells.^47^ METTL14 expression is also regulated by transcription factors. In pancreatic cancer, transcription factor P65 positively regulates METTL14 expression by interconnecting with the promoter region of METTL14. Upregulated METTL14 hampers the attenuation of cytidine deaminase (CDA) transcript, enhances its stability, and induces chemotherapy resistance of pancreatic cancer cells.^48^ Non‐coding RNAs (ncRNAs) also play a role in regulation of METTL14 expression. In breast cancer, the stability and expression of METTL14 are positively regulated by LNC942, which elevates the m6A content in downstream targets CXCR4 and CYP1B1, stabilizes protein expression and translation, and further promotes tumorigenesis.^49^ The expression of METTL14 is regulated by miR‐103‐3p to inhibit osteogenesis, and m6A modification executed by METTL14 in turn promotes osteogenesis by inhibiting the treatment of miR‐103‐3p by DiGeorge critical region 8 (DGCR8), which reveals the key role of miR‐103‐3p ‐ METTL14 ‐ m6A modification axis in osteoblast activity.^50^ In vascular endothelial cells, the expression level of METTL14, modulated by miR‐4729, is reduced, resulting in declination in m6A modification level of TIE1 mRNA 3′UTR specific site, TIE1 mRNA stability reduction, and angiogenesis inhibition.^51^ In renal cell carcinoma, METTL14 is regulated by the competitive binding effect of circRNAs and miRNAs, and affects the expression of downstream molecule PTEN and changes in related AKT/PKB signals.^52^ In skin tumors, METTL14 acts as the target of NBR1‐dependent selective autophagy, and its expression level is down‐regulated. METTL14 depletion reduces the translation efficiency of damaged DNA binding protein 2 (DDB2), causes global genome repair (GGR) repair defect, and provokes UVB‐induced skin tumorigenesis.^53^ In Epstein‐Barr virus (EBV)‐related cancers, METTL14 expression and stability is upregulated by Epstein‐Barr nuclear antigen 3C (EBNA3C) through interaction at the specific amino domain of EBNA3C.^54^

## 4 METTL14 AND CANCER

In recent decades, increasing investigations have shown that METTL14, as an m6A methyltransferase, is involved in tumorigenesis and development, whether as an oncogene or an anti‐oncogene, as shown in Table 1.

**TABLE 1:** METTL14 acts as anti‐oncogene and oncogene in human cancers

Role	Cancer type	Regulator	Targets	Molecular mechanism	Signaling pathway/Axis	Ref.
Anti‐oncogene	Bladder cancer		Notch1	mRNA stabilization	METTL14 ‐ m6A modification ‐ Notch1 axis	^55^
	Colorectal cancer		SOX4	mRNA degradation	METTL14 ‐ YTHDF2 ‐ SOX4 axis; SOX4‐mediated EMT process and PI3K/AKT signaling pathway partly	^56^
		DGCR8	miR‐375	Modulate pri‐miR‐375 process in an m6A‐dependent manner	METTL14 ‐ miR‐375 ‐ YAP1 axis and METTL14 ‐ miR‐375 ‐ SP1 axis	^57^
			lncRNA XIST	LncRNA degradation	METTL14 ‐ YTHDF2 ‐ lncRNA XIST axis	^58^
		MeCP2	KLF4	mRNA stabilization	MeCP2 ‐ METTL14 ‐ KLF4 axis	^75^
	Endometrial cancer		PHLPP2; mTORC2	Promote translation by YTHDF1; mRNA degradation by YTHDF2	METTL14‐ PHLPP2/mTORC2‐AKT pathway	^60^
	Gastric cancer		Wnt and PI3K signals	Regulate Wnt and PI3K/AKT/mTOR pathway	Wnt and PI3K/AKT/mTOR signaling pathways	61, 79
			LINC01320	LncRNA stabilization	METTL14 ‐ LINC01320 ‐ miR‐495‐5p ‐ RAB19 axis	^62^
			PTEN	mRNA stabilization	METTL14 ‐ m6A modification ‐ PTEN axis	^80^
	Glioblastoma		ADAM19	mRNA m6A modification	METTL14 ‐ m6A modification ‐ ADAM19 axis	^63^
	Hepatocellular carcinoma	DGCR8	miR‐126	Modulate pri‐miR‐126 process in an m6A‐dependent manner	DGCR8 ‐ METTL14 – miR‐126 axis	^64^
			USP48	mRNA stabilization	METTL14 ‐ USP48 ‐ SIRT6 axis	^65^
			EGFR	Stimulate PI3K/AKT signals	EGFR/PI3K/AKT signaling pathway	^66^
	Renal cell carcinoma		P2RX6	pre‐mRNA splicing	ATP‐P2RX6‐Ca2+‐p‐ERK1/2 ‐MMP9 signals	^68^
		circRNAs	PTEN	mRNA stabilization	circRNAs ‐ miRNAs ‐ METTL14 ‐ PTEN axis	^52^
	Papillary thyroid carcinoma		OIP5‐AS1	Regulate expression	EGFR, MEK/ERK signaling pathway	^86^
	Osteosarcoma		Caspase‐3	Activate caspase‐3	Apoptosis	^87^
	Skin tumor	NBR1‐dependent autophagy	DDB2	Translation efficiency	NBR1‐dependent autophagy ‐ METTL14 – DDB2 axis	^53^
Oncogene	Acute myeloid leukemia	SPI1	MYB; MYC	Accelerate hematopoietic stem cells proliferation and reduce monocyte differentiation	SPI1 ‐ METTL14 ‐ MYB/MYC axis	^46^
		MALAT1	PML‐PARα	mRNA exportation	lncRNA ‐ fusion gene ‐ METTL14 loop	^92^
	Breast cancer	LNC942	CXCR4; CYP1B1	mRNA stabilization and translation	LNC942 ‐ METTL14 ‐ CXCR4/CYP1B1 axis	^49^
		AURKA	DROSHA	mRNA stabilization	AURKA ‐ METTL14 ‐ DROSHA axis	^47^
	Pancreatic cancer		PERP	mRNA turnover	METTL14 ‐ m6A modification ‐ PERP axis	^95^
		P65	CDA	mRNA stabilization	P65 ‐ METTL14 ‐ CDA axis	^48^
	Prostate cancer	CLK1‐SRSF5 axis		Alternative splicing	CLK1 ‐ SRSF5 ‐ METTL14^exon10^ skipping axis	^97^
	Head and neck squamous cell carcinoma		LNCAROD	LncRNA stabilization	METTL14 – LNCAROD ‐YBX1/HSPA1A axis	^99^
	EBV‐related tumors	EBNA3C		Maintain METTL14 protein stability		^54^

## 5 METTL14 AS AN ANTI‐ONCOGENE

In most cases, METTL14 is identified as a tumor suppressor gene, which inhibits oncogenesis and progression of various cancers, such as bladder cancer,^55^ colorectal cancer,^56^, ^57^, ^58^ endometrial cancer,^59^, ^60^ gastric cancer,^61^, ^62^ glioblastoma,^63^ hepatocellular carcinoma,^64^, ^65^, ^66^ and renal cell carcinoma,^52^, ^67^, ^68^ by reducing m6A modification on key transcripts.

### 5.1 Bladder cancer

Bladder cancer is a widespread malignant cancer worldwide.^69^ Bladder cancer stem cells (CSCs), a subgroup of bladder cancer cells also known as tumor‐initiating cells (TICs), can self‐renew and differentiate into large cell populations, and are rich in tumorigenic properties.^70^ Gu et al.^55^ found that METTL14 expression is reduced in bladder cancer and bladder TICs, and it is the main regulator for the decline of m6A content in bladder cancer and bladder TICs. METTL14 reduction promotes cell propagation, aggression, and self‐renewal of bladder TICs. Studies have shown that neurogenic locus notch homolog protein 1 (Notch1) plays a part in accelerating bladder cancer and bladder TICs. The m6A modification of Notch1 is reduced after overexpression of METTL14, which in turn depresses the stability of Notch1 mRNA and inhibits protein expression. The above results indicate that METTL14 may target Notch1 to inhibit progression of bladder cancer and spread of bladder TICs, which reveals that the METTL14‐m6A‐Notch1 axis has the potential to serve as an underlying target for the treatment of bladder cancer.

### 5.2 Colorectal cancer

Colorectal cancer (CRC), the third most prevalent digestive tract malignancy worldwide, has seen an increasing incidence rate of CRC in the younger population.^71^, ^72^ The expression of METTL14 is remarkably depressed in CRC, and a reduction in METTL14 is associated with poor overall survival (OS). Cox regression analysis has revealed that METTL14 is an independent prognostic molecule for CRC, and it is positively associated with the level of immune infiltration.^73^ It was proved that METTL14 impedes the metastasis of CRC cells and exerts a tumor suppressor effect. Studies have found that SRY‐related high‐mobility‐group box 4 (SOX4) is controlled by m6A modification mediated by METTL14, and METTL14 represses the progression of CRC via the SOX4‐mediated EMT process and PI3K/AKT signals.^56^ It was found in another study that METTL14, as the upstream target of miR‐375, suppresses CRC cells propagation via the miR‐375—Yes‐associated protein 1 (YAP1) axis, and depresses migration and aggression of CRC cells via the miR‐375 ‐ SP1 axis.^57^ Yang et al.,^58^ using RNA sequencing (RNA‐seq) and methylated RNA immunoprecipitation (Me‐RIP), found that the oncogene lncRNA XIST is the downstream molecule of METTL14, which down‐regulates lncRNA XIST in an m6A‐dependent process and inhibits tumor‐driving effects such as the growth and metastasis of CRC. In CRC that is resistant to immunotherapy, mismatched repair proficiency or low microsatellite instability (pMMR‐MSI‐L) tumors account for about 85% of all patients. Studies have proved that reducing m6A modification by knocking down METTL14 escalates the response of pMMR‐MSI‐L CRC to anti‐PD‐1 treatment.^74^ METTL14 and methyl CpG binding protein 2 (MeCP2) jointly regulate the m6A modification of the specific site of the tumor suppressor Kruppel‐like factor 4 (KLF4) mRNA through the protein interaction between the two proteins. The M6A reader IGF2BP2 regulates the stability of KLF4 mRNA and the expression of KLF4 protein by reading unique methylation modification information, and ultimately affects the progression and metastasis of CRC.^75^

### 5.3 Endometrial cancer

Endometrial cancer (EC) is a common gynecologic malignancy, originating from endometrium that grows out of control.^76^, ^77^ Ma et al.^59^ described METTL14 as a probable indicator for the diagnosis and prognosis of EC. Liu et al.^60^ sequenced clinical specimens of EC and found that METTL14 has a hot spot R298P mutation, which induces about 70% of EC to reduce m6A methylation compared with matched normal endometrium, contributing to the proliferation of EC cells and tumorigenicity. Analysis of m6A‐seq characteristics of EC samples from patients and cell lines shows that the decline in m6A mRNA methylation motivated by the METTL14 mutation may cause a decreased level of the negative effector PHLPP2 of the AKT signals and an increase in the level of the positive effector mTORC2, resulting in increased AKT activity and enhanced cell proliferation. METTL14 was found to be an important governor of the AKT signals and cell propagation in EC.

### 5.4 Gastric cancer

Gastric cancer (GC), also known as stomach cancer, is a leading digestive system cancer worldwide and still contributes to human death in less developed countries.^78^ METTL14 is lowly expressed in GC and is a key regulatory factor that decreases m6A levels in GC.^61^ METTL14 overexpression curbs the propagation of GC cells by inhibiting Wnt and PI3K/AKT/mTOR signals, and suppresses aggression of gastric cancer cells by obstructing the EMT process, while an increase in m6A caused by FTO knockdown reverses the above changes.^61^, ^79^ Hu et al.^62^ demonstrated that METTL14 methylates LINC01320 to upregulate LINC01320 expression, accelerating aggression of GC cells through the miR‐495‐5p‐RAB19 axis. Yao et al.^80^ verified that METTL14 enhances phosphatase and tensin homologue (PTEN) m6A modification by interconnecting the m6A characteristic sequence GGACT with PTEN mRNA, improving RNA stability of PTEN and playing a role in cancer inhibition in stomach adenocarcinoma (STAD).

### 5.5 Glioblastoma

Glioblastoma (GBM), occurring predominantly among adults, is the most common and aggressive type of glioma.^81^ METTL14 deficiency considerably accelerates the propagation, self‐renewal, and oncogenesis of glioblastoma stem cells (GSCs). After METTL14 knockdown GSCs transplanted into immunodeficiency non‐obese diabetes/severe combined immunodeficiency (NOD/SCID) mice, it was found that METTL14 depletion results in significant tumor progression initiated by GSCs in the cerebra of the mice. The results of m6A sequencing showed that METTL14 knockdown leads to overexpression of the oncogene ADAM19 mRNA in GSCs.^63^ It implies that METTL14 and m6A modification may have the potential to be a brand‐new molecular target for GBM treatment.

### 5.6 Hepatocellular carcinoma

Hepatocellular carcinoma (HCC), accounting for more than 80% of primary liver carcinoma, is the most common malignant carcinoma of liver cells and is an urgent health threat worldwide.^82^, ^83^ In hepatocellular carcinoma (HCC), particularly in metastatic HCC, m6A modification is reduced, METTL14 is identified as the core molecule regulating abnormal m6A modification of HCC. In addition, down‐regulation of METTL14 is a detrimental prognostic factor for recurrence‐free survival of HCC and is appreciably related to tumor aggression. Furthermore, miR‐126, as an anti‐oncogene, is the target of METTL14 in the process of HCC metastasis. METTL14 interacts with DGCR8 and regulates the pri‐miR‐126 process to reduce miR‐126 expression, thereby promoting HCC metastasis, which accounts for an important function of METTL14 and m6A in HCC metastasis.^64^ Du et al.^65^ found that ubiquitin‐specific peptidase 48 (USP48) bound SIRT6 through deubiquitination at specific sites of SIRT6 and stabilized SIRT6 mRNA, thereby hindering metabolic reprogramming to inhibit HCC oncogenesis. Importantly, METTL14 stabilizes USP48 mRNA by provoking m6A modification. The METTL14‐USP48‐SIRT6 axis is involved in glycolysis regulation to suppress HCC, and hence is considered a promising target for future therapeutic research. Shi et al.^66^ found by overlapping RNA‐seq and m6A‐seq that EGFR is the downstream target of METTL14 in HCC. EGFR is an important oncogene that is present in most solid cancers and is significantly pertinent to the stimulation of the PI3K/AKT signals in HCC. Under‐expressed METTL14 upregulates EGFR expression in an m6A‐dependent manner, contributing in activating the PI3K/AKT signals and enhancing the propagation of HCC cells. It has been proved that METTL14 hampers oncogenesis and metastasis of HCC cells by regulating the EGFR/PI3K/AKT signals.

### 5.7 Renal cancer

METTL14 is predominantly positioned in the nucleus of clear cell renal cell carcinoma (ccRCC) cells. Compared with normal renal tissues, the ccRCC tissues have a significantly lower level of METTL14 mRNA. The expression level of METTL14 is positively correlated with OS, and is also related to the clinicopathological factors of RCC (sex, T stage, M stage, and pathological stage).^84^, ^85^ Studies have found that the VHL‐HIF‐ZNF217‐METTL14‐mediated hypoxia pathway affects generation and sustentation of the RCC cancer stem cells by regulating m6A levels, and mediates oncogenesis and development of ccRCC via the PI3K/AKT/mTOR and p53 signaling pathways that are the downstream targets of m6A.^67^ In normal renal tissues, METTL14 elevates the pre‐mRNA splicing of P2RX6 by upregulating the m6A content of P2RX6 mRNA, leading to a P2RX6 reduction. In RCC cells, however, METTL14 depletion results in the high expression of P2RX6 via the ATP‐P2RX6‐Ca2+‐p‐ERK1/2‐MMP9 signaling pathway and finally accelerates the aggressiveness of RCC cells.^68^ In addition, METTL14 mediates stability of PTEN mRNA that negatively regulates the AKT/PKB signaling pathway and exerts a tumor suppressor effect. METTL14 also interacts with EIF3A to generate a collaborative effect and modulate the tumorigenesis of RCC.^52^ Using bioinformatics analysis, Wang et al.^52^ found that the level of METTL14 in RCC presents significantly negative correlation to the expression of four miRNAs, miR‐130a‐3p, miR‐106b‐5p, miR‐130b‐3p, and miR‐301a‐3p, which regulate the METTL14 expression by interacting with a series of 24 circRNAs, such as circ‐0023414 and circ‐0031772.

### 5.8 Other cancer

In papillary thyroid carcinoma (PTC), OIP5‐AS1 is a downstream target of METTL14. METTL14 upregulation hinders the expression of OIP5‐AS1, regulates EGFR, MEK/ERK signals, and depresses PTC cell proliferation and aggression.^86^ METTL14 exerts a tumor suppressor effect by activating caspase‐3 to constrain the propagation, migration, and aggression of osteosarcoma cells, and is considered a potential target for osteosarcoma treatment.^87^

## 6 METTL14 AS AN ONCOGENE

Although METTL14 has a tumor suppressor effect in most cancer types, it has also been found to act as a contributing factor to tumorigenesis and development.

### 6.1 Acute myeloid leukemia

Acute myeloid leukemia (AML), characterized by aberrant clonal expansion and impaired differentiation of hematopoietic stem and progenitor cells (HSPCs), is the most common form of acute leukemia among adults.^88^, ^89^ Weng et al.^46^ identified the position of METTL14 in tumorigenesis and progression of AML. Analysis of TCGA data showed that METTL14 is highly expressed in bone marrow mononuclear cells (MNCs) of AML patients and of primary AML patients with common chromosomal translocation such as *AML1‐ETO* (t8; t21), *PML‐RARα* (t15; t17), and *MLL* fusions (t11). However, METTL14 is down‐regulated in directed or differentiated myeloid cells during myeloid differentiation. In experiments, METTL14*^fl/fl^*‐CRE^ERT^ conditional knockout mice (METTL14 flox (METTL14*^fl/+^*) generated by CRISPR/Cas9 technique were crossbred to produce homozygous METTL14 knockout mice (METTL14*^fl/fl^*). The genetic switch is the CRE^ERT^ system consisting of three key components—CRE recombinase, ER (estrogen receptor), and T (Tamoxifen). Briefly, the CRE^ERT^ system can be induced to turn on by the drug tamoxifen. To eliminate the effect of endogenous estrogen, the estrogen receptor of the mouse was mutated. The mutated estrogen receptor no longer binds to estrogen and has an affinity only for foreign drugs like tamoxifen. In other words, only when tamoxifen appears can the CRE enzyme enter the nucleus to regulate gene expression.^90^, ^91^ The experiments on the mice show that METTL14 depletion inhibits the proliferation and self‐renewal activity of HSCs in vivo. The above results indicate that METTL14 promotes leukemia progression by accelerating HSC proliferation and renewal, and by reducing monocyte differentiation. Importantly, METTL14 is adversely modulated by SPI1 and positively mediates the expression of MYB and MYC via m6A‐dependent post‐transcriptional modulation. The above results reveal that the SPI1‐METTL14‐MYB/MYC axis plays a key role in the formation of malignant myelopoiesis and the pathogenesis of AML. In addition, METTL14 can be directly combined with MALAT1. Importantly, the interaction between METTL14 and fusion‐gene PML‐RARα is regulated by MALAT1, a functional loading bridge, which further elevates PML‐RARα mRNA METTL14‐dependent m6A modification and accelerates the fusion‐gene exporting. The lncRNA‐fusion‐gene‐m6A loop regulates the differentiation of myeloid cells by controlling the export of the fusion gene and affects the malignant hematopoietic process.^92^

### 6.2 Breast cancer

Breast cancer (BC), the most frequent female malignant neoplasia worldwide, is in want of better prognosis solutions.^78^, ^93^ Yi et al.^94^ observed upregulation of METTL14 in breast cancer tissues and its correlation to the TNM stage of patients. Specifically, high expression of METTL14 changes the miRNA expression profile and increases the expression of hsa‐miR‐146a‐5p, thereby promoting migration and aggression of BC cells. Additionally, in BC cells and tissues, METTL14 mRNA stability and protein expression are positively modulated by LNC942. LNC942 and METTL14 protein specifically bind through specific motif/GCAGGG (+176 – +265) to elevate the m6A content of downstream target molecules such as CXCR4 and CYP1B1, thus stabilizing the expression and translation of CXCR4 and CYP1B1, and ultimately promoting BC cell propagation in vivo and in vitro.^49^ In breast cancer stem‐like cells, METTL14 mRNA stability and protein expression are upregulated by overexpression of the upstream molecule AURKA, which stabilizes METTL14 protein by inhibiting ubiquitylation‐dependent degradation of METTL14, resulting in enhanced DROSHA mRNA stability through METTL14‐dependent m6A modification. More importantly, binding of AURKA to DROSHA transcript further strengthens the binding of IGF2BP2 to the m6A modified transcript, hence increasing DROSHA mRNA stability and ultimately enhancing BCSC phenotype.^47^

### 6.3 PANCREATIC CANCER

Wang et al.^95^ found increased m6A modification in more than half of pancreatic cancer tissues, and identified METTL14 as the major molecule that regulates m6A methylation in pancreatic cancer. Specifically, overexpressed METTL14 directly targets the downstream PERP mRNA to enhance propagation and migration of pancreatic cancer cells in an m6A‐dependent way. Kong et al.^96^ validated that down‐regulation of METTL14 makes pancreatic cancer cells sensitive to cisplatin by accelerating apoptosis, and improves autophagy induced by cisplatin through the mTOR signaling‐dependent pathway. Another study elucidated the mechanism of METTL14 in drug resistance in pancreatic cancer. In gemcitabine‐resistant pancreatic cancer cells, METTL14 is regulated by P65, a transcriptional factor binding to METTL14 promoter region, and elevates the transcript stability of CDA, which causes gemcitabine inactivation, and ultimately developments gemcitabine resistance of pancreatic cancer cells in vitro and in vivo.^48^ These results suggest that METTL14 may possess feasibility in treating chemotherapy‐resistant pancreatic cancer.

### 6.4 Prostate cancer

By transcriptome sequencing, Chen et al.^97^ identified METTL14^exon10^ and Cyclin L2^exon6.3^ skipping as vital alternative splicing events managed by the CLK1‐SRSF5 axis. The phosphorylation of SRSF5^250‐Ser^, reinforced by CLK1, promotes the skipping of Cyclin L2^exon6.3^ while hampering inhibits the skipping of METTL14^exon10^, which enhances m6A modification and increases the migration capacity of prostate cancer cells. METTL14 upregulation was found to be closely correlated with the recurrence‐free survival of prostate cancer. Specifically, METTL14 overexpression elevates the m6A modification level of mRNA and enhances the advancement of prostate cancer by managing the localization of subcellular proteins.^98^

### 6.5 Other cancer

METTL14‐mediated m6A improves the stabilization of LNCAROD, an oncogenic lncRNA in head and neck squamous cell carcinoma (HNSCC) cells, and acts as a scaffold in the protein interaction between YBX1 and HSPA1A to maintain the stability of YBX1 protein, and contributes to HNSCC cell propagation, migration, and oncogenesis.^99^ EBV, one of the most prevalent oncogenic viruses, causes a variety of tumors. Lang et al.^54^ found that EBV drives the proliferation of EBV‐transformed cells and related oncogenesis by EBNA3C that upregulates protein levels and stability of METTL14 which interacts with specific amino domains of EBNA3C. METTL14 is considered to be an innovative molecular target for treatment of EBV‐related tumors.

## 7 CONCLUSIONS AND PERSPECTIVES

In conclusion, m6A affects various aspects of RNA physiological processes, such as mRNA processing, translation efficiency, and transcription stability, and contributes to developmental regulation, cell cycle, and other cellular physiological processes of tumors and other diseases. METTL14, as an m6A methyltransferase, regulates m6A contents. Existing works have confirmed that METTL14 plays a pivotal role in many aspects of tumor progression, including cell accumulation, aggression, apoptosis, and self‐renewal, as either an oncogenic and anti‐oncogenic factor. However, the role of METTL14 in tumors and its potential molecular mechanism is far from being fully clarified. It should be noted that, however, the role of METTL3, another important component of MTC, should be considered in investigations on the role of METTL14 in tumors. Theoretically speaking, METTL14 and METTL3, both as components of MTC, play similar roles in the same tumor. This holds true for some cases. For instance, METTL3 was found to act as a tumor suppressor alike METTL14 in tumors such as renal cancer and endometrial cancer, and plays a tumor‐promoting role alike METTL14 in tumors such as AML, breast cancer, and pancreatic cancer. In tumors such as HCC and gastric cancer, however, these two proteins work in opposite ways. The causes for the opposing roles of these two constituents of MTC in the same tumor may be tumor heterogeneity, their different protein structures, and, in some cases, different study models. The discrepancy remains to be investigated further.

Since METTL14 has extensive impacts on tumor progression and plays different roles in various tumors, it is expected to become a novel molecule for tumor diagnosis and treatment, but specific METTL14‐targeted therapy strategies are yet to be explored.

## CONFLICT OF INTEREST

All authors declare that they have no conflict of interest.

## AUTHOR CONTRIBUTIONS

XL conducted research and drafted the manuscript. YPD, ZHH, HLQ, and JWC provided assistance in the process of revised drafting manuscript and figure and tables construction. YZ contributed to conceptual framework, supervised the study, and revised the manuscript. All authors read the final manuscript and approved.

## Data Availability

The data that support the findings of this study are available from the corresponding author upon reasonable request.
